# Real-Time Gait Phase Detection Using Wearable Sensors for Transtibial Prosthesis Based on a kNN Algorithm

**DOI:** 10.3390/s22114242

**Published:** 2022-06-02

**Authors:** Atcharawan Rattanasak, Peerapong Uthansakul, Monthippa Uthansakul, Talit Jumphoo, Khomdet Phapatanaburi, Bura Sindhupakorn, Supakit Rooppakhun

**Affiliations:** 1 School of Telecommunication Engineering, Institute of Engineering, Suranaree University of Technology, Nakhon Ratchasima 30000, Thailand; m6303129@g.sut.ac.th (A.R.); mtp@sut.ac.th (M.U.); drivilmiz@gmail.com (T.J.); 2 Department of Telecommunication Engineering, Faculty of Engineering and Technology, Rajamangala University of Technology Isan (RMUTI), Nakhon Ratchasima 30000, Thailand; khomdet.ph@rmuti.ac.th; 3 Orthopedic Department School of Medicine, Suranaree University of Technology, Nakhon Ratchasima 30000, Thailand; bura@sut.ac.th; 4 School of Mechanical Engineering, Institute of Engineering, Suranaree University of Technology, Nakhon Ratchasima 30000, Thailand; supakit@sut.ac.th

**Keywords:** k-nearest neighbor, wearable sensor, gait analysis, gait phase detection, transtibial prosthesis

## Abstract

Those with disabilities who have lost their legs must use a prosthesis to walk. However, traditional prostheses have the disadvantage of being unable to move and support the human gait because there are no mechanisms or algorithms to control them. This makes it difficult for the wearer to walk. To overcome this problem, we developed an insole device with a wearable sensor for real-time gait phase detection based on the kNN (k-nearest neighbor) algorithm for prosthetic control. The kNN algorithm is used with the raw data obtained from the pressure sensors in the insole to predict seven walking phases, i.e., stand, heel strike, foot flat, midstance, heel off, toe-off, and swing. As a result, the predictive decision in each gait cycle to control the ankle movement of the transtibial prosthesis improves with each walk. The results in this study can provide 81.43% accuracy for gait phase detection, and can control the transtibial prosthetic effectively at the maximum walking speed of 6 km/h. Moreover, this insole device is small, lightweight and unaffected by the physical factors of the wearer.

## 1. Introduction

Gait analysis is widely known and researched because it has benefits in many fields. For example, in the medical field, gait analysis is often used to look for various diseases caused by walking disorders [1,2]. In addition, the engineering field can be used to analyze human leg and foot locomotion to detect the gait phase and improve prostheses’ performance [3]. There are two essential things in gait phase detection. The first thing is a method of collecting gait information, and the second thing is a method used to identify the gait phase.

Gait phase detection based on a vision system is a method that uses a camera to analyze body movements during walking. In general, a camera is used to measure the characteristics of hip, knee, and ankle angles. In addition, a vision system is usually used with the force plates to increase the efficiency of detecting the gait phase. Varol et al. presented a study on controlling a transfemoral prosthesis using a kNN algorithm with a predominant weighting scheme and a weighting threshold, using seven cameras and two force platforms. The two force plates measured the ground reaction force to increase the effectiveness of real-time recognition of a person’s walking intent who would stand or walk at one of three different speeds, including slow (~1.04 m/s), normal (~1.22 m/s), and fast (~1.51 m/s) [3]. However, this method is only required in a laboratory or controlled environment, so it is unable to be used in daily life. Therefore, detecting the gait phase should be performed with a wearable device that can easily detect the walking distance.

A wearable device uses an inertial measurement unit (IMU) [4,5,6,7]. There are many techniques for IMU usage [4]: Huang et al. presented a lightweight convolutional neural network (CNN) model with an attention mechanism for detecting gait characteristics using wearable IMU sensors [5]. In addition, Mahoney et al. presented an artificial neural network (ANN) model that classifies the type of stride with an accelerometer and gyroscope (IMU) affixed to the user’s left ankle [6]. Furthermore, Wu et al. demonstrated the feasibility of predicting ground reaction force by using IMU sensors mounted below the walking surface with long short-term memory (LSTM) models [7]. However, errors will occur when using an IMU with speed and location data due to interference and signal drift. Additionally, there are still limitations in terms of training data in machine learning.

One of the most popular ways to detect the gait phase is to have a pressure sensor on the bottom of the wearer’s foot to analyze the ground reaction force (GRF) during walking [8,9,10]. Methods used to identify the gait phase include the center of pressure (COP). The ratio of COP values is used to divide the walking cycle and identify the point of weight movement between the two feet [8]. In addition, a support vector machine (SVM) can identify the gait phase too. The SVM is a neural network for supervised learning. It learns from a sample set of input and output values and can detect discrete walking steps trete withinside the round movement of dynamic human walking [9]. A pressure sensor is sometimes used with IMU to increase detection efficiency. Nagashima et al. predicted plantar force during gait to detect walking distance with a deep neural network (DNN). The DNN is used to learn the non-linear relationship between the measured sensor data and future sensor force data by using a trained network [10]. However, when detecting the gait phase, problems are often found in the wearer’s physical condition, such as the sensor position, stride speed, and weight [11]. In addition, there are still problems with processing and control delays, making the prosthesis control ineffective.

In terms of the machine learning approaches and their performances in the studied papers, most of the papers [12,13] have used kNN as their machine learning approach to classify the gait cycle pattern. Most of these studies compared the output of the kNN algorithm with those of other approaches. In [12], for the classification of three gait patterns through GRFs data, artificial neural network algorithms (ANN) were used for comparison with the k-nearest neighbor approach. kNN showed the best accuracy of 85%, slightly higher than the ANN at 80%. Another paper [13] has shown five-class classification with SVM, decision tree (DT), ANN, naive Bayes (NB), and kNN. The overall classification accuracy was 97.9% for SVM, 90.1% for DT, 100% for ANN, 97.2% for NB, and 100% for kNN. Thus, in this study, we choose the kNN algorithm for gait classification because the kNN outperforms all other approaches in terms of accuracy.

In this paper, we present a k-nearest neighbor algorithm that can detect a real-time gait phase among seven phases, i.e., stand, heel strike, foot flat, midstance, heel off, toe-off, and swing phase, by using a wearable sensor located in an insole sheet which can be worn with all types of shoes. Furthermore, it can control the prosthesis via Bluetooth with little delay. The wearable device small, lightweight, and unaffected by the physical factors of the wearer.

## 2. Materials and Methods

### 2.1. Hardware Description

**Figure 1:**
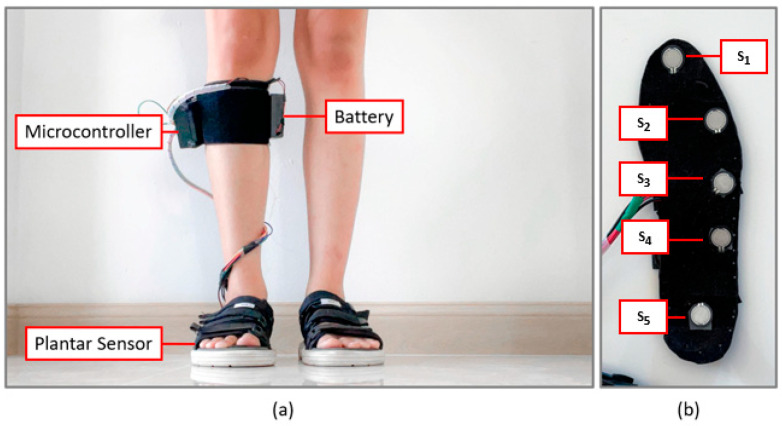
(**a**) The equipment of the wearable sensor device to receive data during walking. (**b**) Five sensors’ positions in the insole sheet.

The insole device developed in this study consists of three main components, as shown in Figure 1a. Firstly, five plantar force sensors (FSR-402, Interlink Electronics, Camarillo, CA, USA) are attached to the bottom of the insole sheet (S_1_–S_5_) to receive the force exerted on the foot as you walk [14,15]. The sensor positions are shown in Figure 1b, though they change according to the shape of the wearer’s foot. Next, notice the ~6 v battery that is the power supply for the device, and lastly, notice the microcontroller board which is used to process the force data and send each force data to the computer for further analysis. We put these three components on a right leg, as shown in Figure 1a.

The first step is the procedure for collecting the five sensor values during human walking. The sensor values are transmitted using wires through a computer. It is necessary to collect data with wires because using a wireless data storage method would cause a delay in data transmission and might cause the loss of essential force data. Therefore, it is necessary to collect the sensor value data with wires, and a complete set of parameters is required to observe each sensor’s rate of change. A wire we used was a micro-USB type B connecting to USB 2.0 Type-A with a length of 3 m, which was connected to the microcontroller board to send data to the computer.

### 2.2. Participants and Experimental Procedures

**Figure 2:**
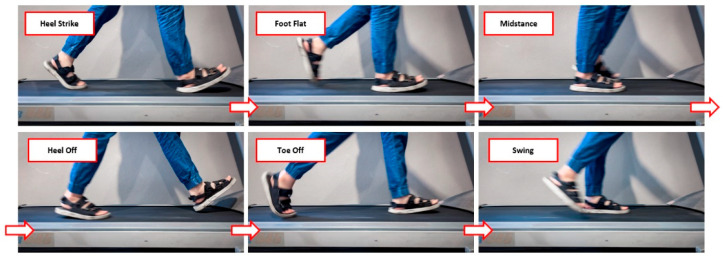
The phases of the gait cycle collected and analyzed in this work.

The gait experiment was conducted on two adult females and three males, 26 ± 3 years of age, who were 165 ± 3 cm in height and 53 ± 2 kg. In terms of recorded data, all participants were tested while standing still for 10 s and then walked on a treadmill at speeds from 1 to 8 km/h. There are seven phases of the gait cycle that we were interested in collecting—stand, heel strike, foot flat, midstance, heel off, toe-off, and swing—as shown in Figure 2. The recorded data were sent to a PC using the JAVA telemetry viewer to collect the sensor values while walking. It can collect 100 sensor values per second.

**Figure 3:**
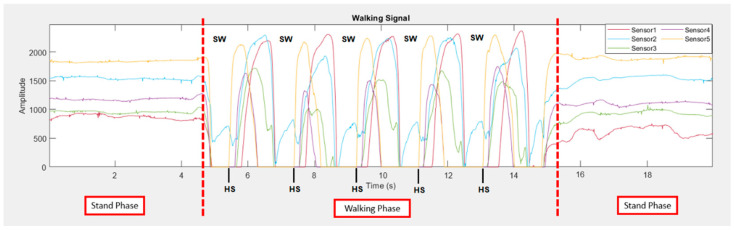
Examples of measured signals from five sensors. (SW: swing, HS: heel strike).

**Figure 4:**
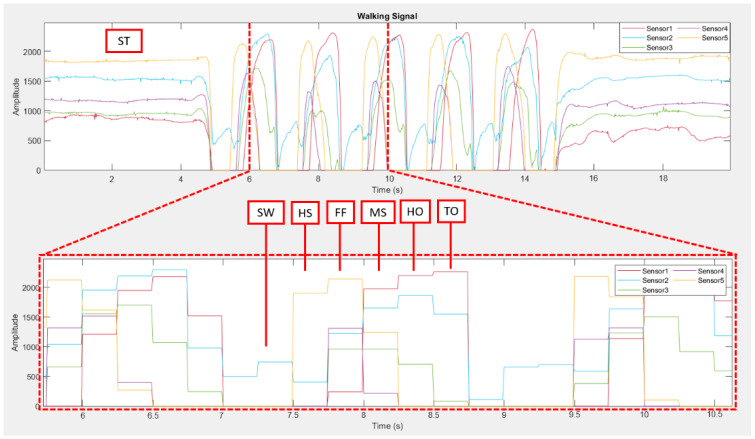
The example of dividing a sensor signal into 7 segments by MATLAB. (ST: stand, SW: swing, HS: heel strike, FF: foot flat, MS: midstance, HO: heel off, TO: toe-off).

Figure 3 shows an example of measured signals from five sensors (S_1_–S_5_), which are raw force sensor signals, unfiltered. During the walking test, it can be seen that each sensor received the same pattern in each walking cycle. The collected force sensor signal was analyzed and filtered through MATLAB to divide the force sensor signal into seven segments. The seven segments are the seven phases of the walking cycle that we were interested in collecting. We divided the sensor signal using a sampling rate of 20 Hz, and we performed the mean range of the data at each sampling to divide each data into seven segments, as seen in Figure 4. After filtering through MATLAB, some data were used as a training dataset labeled in the machine learning kNN, and some data were used to find the best k value that is most suitable for our algorithm. We used the Arduino IDE and the classified intent onboard ESP-WROOM-32 with Bluetooth 4.2 MCU module and 16 Mb flash memory. This work obtained the ethics committee approval from the human research from Suranaree University of Technology (License EC-64-30 COA number 67/2564). This work was supported by Suranaree University of Technology (SUT), Thailand Science Research and Innovation (TSRI), and the National Science Research and Innovation Fund (NSRF) (NRIIS number 42852), and all participants provided written consent for this study prior to participation.

### 2.3. K-Nearest Neighbor Classification

K-nearest neighbor (kNN) classification is one of the simplest machine learning algorithms based on the supervised learning technique. It is widely used in data classification, pattern recognition, picture classification, and other fields [16]. The kNN algorithm stores the entire training dataset and reclassifies the data points according to the distance between every two points for k neighbors using the Euclidean distance function. This distance function is often modified by scaling the attribute so that the spread of the attribute values across dimensions is approximately the same. This formula can calculate the closeness of all training data points to the data points one is trying to label and use the mean of the nearest neighbor k to predict as expressed in (1).

(1) $$Dist\left(p,q\right)=Dist\left(q,p\right)=\sqrt{\overset{n}{\underset{i=1}{\sum }}{\left(q_{i}-p_{i}\right)}^{2}}\;$$

One of the many issues that affect the performance of the kNN algorithm is the choice of the hyperparameter k. If k is too small, the algorithm would be more sensitive to overfitting data points. If the value of k is too large, then the model will be inflexible. For example, if k = N (the total number of data points), the model will just dumbly blanket-classify all the test data as the mean of the training data. Moreover, the number of k should be odd to avoid decision problems in the case of two equal classes.

In this study, we collected walking data using five pressure sensors (S_1_–S_5_) to train in the k-nearest neighbor algorithm. The training data has seven groups named stand, heel strike, foot flat, midstance, heel off, toe-off, and swing. Each class has 70 labeled training sets, so the total is 490 labeled training sets. Additionally, we performed experiments to see which k value is the most suitable for our algorithm and provides the most effective prediction.

### 2.4. Distance Weighted kNN Algorithm

A distance weighted kNN algorithm or weighted voting is weighted based on calculated similarity. This means that more similar data have a more significant impact on answer formulation than less similar data, solving the problem of prediction errors due to *k* being too high or too low. As defined in the equations below, it makes the computational model more accurate. Each of our scores has to be weighed in each class. For neighbor *k*, we sort the distances in ascending order, e.g., d_1_, …, d*_k_*, where d_1_ is the smallest distance, d*_k_* is the furthest distance, and d*_i_* is the original distance of each point. Then, whatever type of total weights the most, let the test point be that class.

(2) $$w_{i}=\left\{\begin{array}{c} \frac{d_{k}-d_{i}}{d_{k}-d_{1}}\;\;\;if\;k\neq 1\\ 1\;\;\;\;\;\;\;\;\;\;if\;k=1 \end{array}\right.$$

After completing the implementation of this equation, a new distance weighted value of each class will be acquired, and then we can see which group or which class has the largest distance weighted value. Finally, the algorithm predicts that the test point is of that class. Thus, in this paper, we used this distance-weighted kNN to help in terms of improving predictive performance and making walking cycle detection better. Moreover, using a distance-weighted k-nearest neighbor can reduce the number of errors during prediction and make the kNN algorithm work even faster.

### 2.5. Calibration System

In research analysis of gait cycles or gait phases, physical problems of the wearer are often identified, such as the wearer’s weight affecting the gait cycle detection. This is because most of the algorithms rely primarily on the force of the sensor to detect the gait phase correctly. The weight of the wearer affects the strength of the sensor, and may cause false detection [17,18]. We have added a calibration system to our wearables to solve this problem. It automatically adjusts the weight on the sensor at the touch of a button.

**Figure 5:**
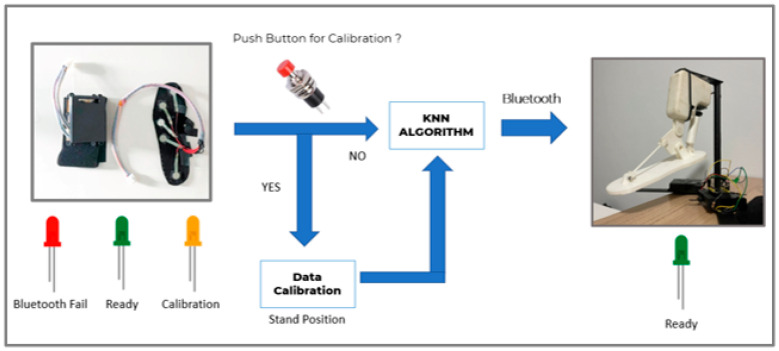
The schematic of the gait analysis with the calibration system for controlling the transtibial prosthetic.

The schematic of the gait analysis system with a calibration system for controlling the transtibial prosthetic is illustrated in Figure 5 Our devices have LED lights that show all three working states. A red LED means that our wearable device is still unable to connect via Bluetooth with the prosthetic foot. The green LED means the wearable device is successfully connected to the prosthetic foot, and the orange LED indicates that the calibration is in progress. In the process of calibration, when the switch is turned on, the wearer must be in the standing position and press the button. Then, the orange LED is turned on, which means the calibration system is working. Then, the wearer waits until the orange LED turns green. Then, the wearer can start walking. The data of each force sensor are taken to the k-nearest neighbor algorithm to predict the gait cycle phase. Then, the predicted data are transmitted to the prosthetic foot for control via a Bluetooth connection.

## 3. Results and Discussion

The experiments were conducted on a self-collected dataset that consists of the sensor values gathered from five individuals. One-hundred and forty samples were created for a single person. This, there are in total 700 samples. We split the data into training and testing sets. The training set has 70% of the complete data (490 labeled training sets), and the testing set has 30% of the dataset (210 labeled training sets). Then, each sensor value was analyzed and taken into the kNN process to predict seven gait phases, i.e., stand, heel strike, foot flat, midstance, heel off, toe-off, and swing, using the Euclidean distance function.

The experimental results can be divided into four essential parts. First is the optimal k value for our kNN algorithm, find each gait stage’s classification accuracy with optimal k, the walking speed that affected the accuracy of kNN prediction, and the time delay for controlling transtibial prosthesis via Bluetooth connection.

**Figure 6:**
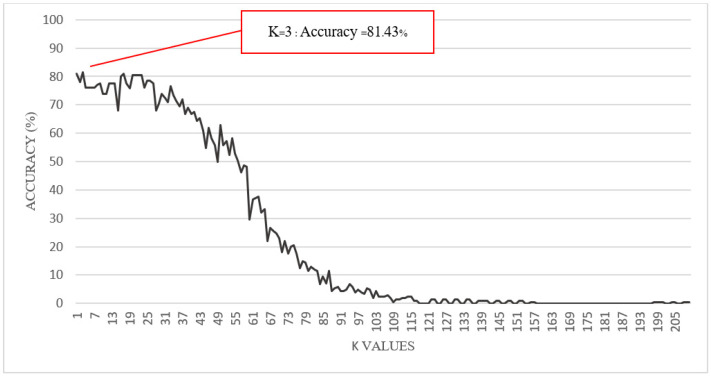
Prediction accuracy of the kNN algorithm with different k values.

Figure 6 shows the prediction accuracy result of the kNN algorithm with different k values. As seen in Figure 6, the selected k value significantly impacts the accuracy of the gait phase prediction in the k-nearest neighbor algorithm. In the beginning, the value of k = 1 had a prediction accuracy of 80.95%, k = 3 had a prediction accuracy of 81.43%, and k = 5 had a prediction accuracy of 76.19%. After that, it can be seen that the larger the k value, the lower the prediction accuracy. Hence, the most suitable value of k in the k-nearest neighbor algorithm is k = 3. That means our kNN algorithm will retrieve every three data points closest to the test point by using the Euclidean distance equation.

Table 1 shows the gait classification accuracy for each gait phase using k = 3. It can be seen that the swing phase had the highest forecast accuracy (100.00%) because the swing phase is distinguished by its distinct signal. The feet are elevated in the swing action, and just a small amount of force is imparted to the sensors at the bottom of the foot. That means the sensors in S_1_, S_2_, S_3_, S_4_, and S_5_ will be subjected to a small amount of force. As a result, the swing phase is simple to learn and has high prediction accuracy via machine learning.

The second highest prediction accuracy was a tie for heel strike and toe-off, for which we achieved 93.34%. Both phases are equally accurately predicted because both have the same sensor value characteristics, just in different positions. In other words, both the heel strike phase and the toe-off phase have similar sensor signal formats, but the heel strike phase is a gesture of placing weight on the heel, so the signal strength of S_5_ is noticeably higher than that of the other sensors (S_5_ >>S_1_, S_2_, S_3_, S_4_). Similarly, the toe-off phase is a gesture of putting weight on the toe, causing the signal strength of S_1_ to be noticeably higher than the signal strength of the other sensors (S_1_ >>S_2_, S_3_, S_4_, S_5_).

The stand and midstance phase prediction accuracies were 70.00% and 66.67%, respectively. This is because these two phases have the same characteristics, e.g., foot position, ankle angle, and force plantar load. Moreover, they have the same force acting on the S_1_ ≈ S_2_ ≈ S_3_ ≈ S_4_ ≈ S_5_. For this reason, machine learning finds it difficult to predict these phases because the two datasets are similar to each other. However, since both phases have the same angle of motion of the ankle, it does not affect the algorithm in the control of prosthetic feet.

The last are foot flat and heel off phases. Both phases had a prediction accuracy of 73.33%. As humans are very fast walkers, both phases are part of the junction phase between the primary phase. The main phases are heel strike, midstance, and toe-off, but the foot flat phase is the junction phase between heel strike and midstance. Similarly, the heel off phase is the junction phase between midstance and toe-off. The wearer’s walking speed increasing may cause cross-phase prediction of these two junction phases to occur.

Cross-phase prediction is one of the error predictions. Predicting the phases in the gait cycle is usually done in the following order: heel strike, foot flat, midstance, heel off, toe-off, and swing. “Cross-phase prediction” is predicting the gait cycle phases earlier than the actual phase. For example, if now it is heel strike phase and the next phase that should be predicted is foot flat, the algorithm could predict midstance instead. It can be seen that the algorithm predicts one phase too early, which is called cross-phase prediction.

Overall, the predictive accuracy of each phase of human gait is an essential variable in prosthetic foot control because the prosthesis is designed to control the angle of the ankle to move up or down according to the phase that machine learning can predict. The highest prediction accuracy of each phase will make the prosthetic foot control be more smooth and more effective. However, suppose the prediction is low accuracy, or there is a prediction error or cross-phase prediction. In that case, the prosthetic foot will not be able to move as it should and will make the wearer of this prosthetic leg unable to walk easily. In the overall results of our individual gait classification accuracy, each phase has a prediction accuracy within the range that the prosthesis could be efficiently controlled without problems.

Nevertheless, the walking speed will affect the prediction performance of the kNN algorithm. Walking speed is one of the major issues in research detecting gait events [19,20,21,22]. Most algorithms tend to cross-predict or are unable to predict at all. Therefore, in this work, we focus on real-time gait phase detection research using wearable sensors for the transtibial prosthesis that can only support normal human walking or approximately 1.4 m/s.

**Figure 7:**
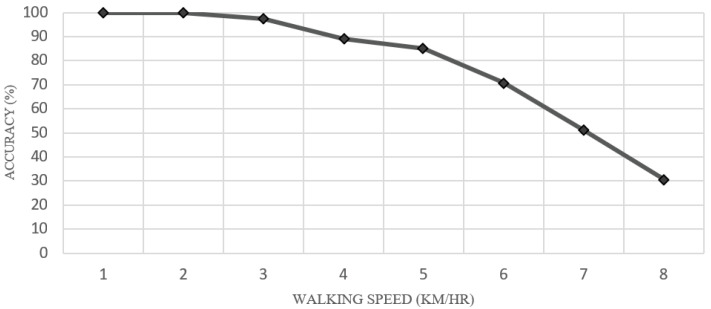
Walking speed affects the prediction accuracy of the kNN algorithm.

In the experiment on walking speed, we tested the accuracy of predicting gait cycles at different speeds by having subjects walk on a treadmill. The speed ranged from 1 to 8 km per hour. The experimental results are shown in Figure 7. It can be seen that the accuracy decreases as the walking speed increases. The walking speed is divided into three parts: slow (1–2 km/h), normal (3–5 km/h), and fast walking (6–8 km/h). First of all, during slow walking, predicting gait cycles was very accurate, in the range of about 90.00–100.00%, because when walking at a slower speed, the gait cycle phase change will also be slow. Furthermore, sensors at the bottom of the foot can perfectly capture the pressure of each gait cycle. This enables machine learning to process and predict gait cycles efficiently.

**Figure 8:**
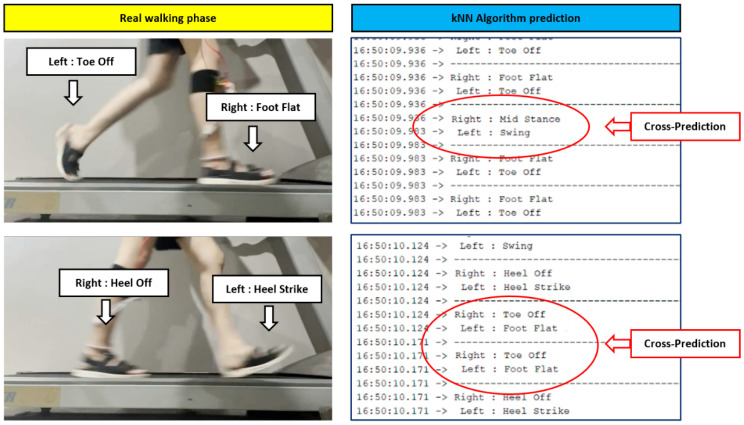
Cross-phase prediction of walking speed at 6 km/h.

The next phase covers the range of normal human walking speeds. As seen in Figure 8, the results show that there is still high prediction accuracy during this normal walking, but worse than for slow walking. The accuracy of this range is in the range of 85.14–97.43%. For the fast walking speed, the accuracy is 30.71–70.81%. The accuracy in this range of walking speeds is significantly worse compared with slow and normal walking. At a walking speed of 6 km/h, the prediction accuracy is just 70.81% because the model can predict only primary phases, such as heel strike, midstance, heel off, stand, and swing; and much cross-phase prediction occurs, e.g., of foot flat and heel off phases.

The kNN algorithm results in cross-prediction of foot flat and heel off phases. This will not have much impact on transtibial prosthetic control because the primary phase is still predictable. Foot flat and heel off phases just will help control the transtibial prosthesis for smooth flow of walking. When the walking speed reaches 7–8 km/h, it can be seen that the prediction accuracy gradually decreases. As at such speeds, the kNN algorithm predicts foot flat and heel off phases incorrectly, including wrongly predicting the main phases, which would significantly affect foot control. As a result, the prosthetic foot could not move according to the gait phases as it should. From the previous results, it can be concluded that walking speed affects the gait cycle prediction algorithm. The faster the wearer walks, the more errors in the predictions [23,24]. As a result, the kNN algorithm in this paper can control the prosthetic foot effectively at up to 6 km/h.

Figure 8 shows the results of each gait phase prediction at a walking speed of 6 km/h. There were errors at the start of the gait cycle known as cross-phase prediction at this speed. Most likely, the algorithm will begin to predict erroneously in the foot flat phase. Foot flat is often predicted as midstance, and heel off is often predicted as toe-off due to the walking speed. The walking speed makes the sensor installed in the insole area unable to receive the value in that phase immediately. This makes the processing of kNN algorithms through the microcontroller board unpredictable. At this speed, it can be seen that most gait phase predictions are accurate, and the accuracy of gait cycle prediction is very high. However, cross-phase prediction occurs between foot flat and heel off phases at this speed, which are junction phases between the primary phases. That would not affect transtibial prosthetic foot control. For the speed of 7–8 km/h, the kNN algorithm made a lot of prediction errors in multiple phases, including junction and primary phases. That means the kNN algorithm cannot operate at this walking speed and would be unable to control a prosthetic foot. Thus, our algorithm can support a maximum human walking speed of 6 km/h. It can control a prosthesis effectively at that speed.

**Figure 9:**
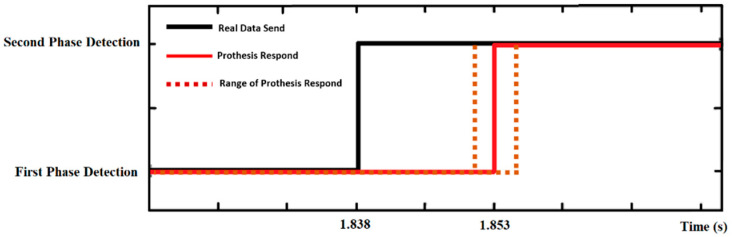
Delay in data transmission via Bluetooth connection for transtibial prosthesis control.

For the real-time implementation—controlling a transtibial prosthesis—two esp-wroom 32 microcontroller boards were used. One was attached to the leg with a wearable device to run the kNN algorithm for phase prediction and send the phase shift data of the gait cycle to the esp-wroom 32, which was attached to the transtibial prosthetic leg to receive data and use the data of phase change to control the prosthesis. We sent the data via Bluetooth connection, causing a delay and the computation time shown in Figure 9. The black line represents when the actual data were sent from the insole device to the prosthesis for control. The red line indicates when the prosthesis responded to the signal sent by the insole device. The brown dashed line represents the range of responses to the signals produced by the prosthetic foot.

With the actual data transmission time and the length of time the prosthesis takes to respond, there is an approximately average delay of 15 ± 2 ms, which is an acceptable delay for receiving data and would not affect the control of the prosthesis.

Excessive Bluetooth transmission delay values can significantly impact prosthetic control if there is too much delay. Although the algorithm is very accurate at predicting gait cycles, the prosthesis could not move according to the prediction of gait cycles if there were a large delay [25,26,27,28]. It will make people with disabilities unable actually to use this prosthetic leg.

## 4. Conclusions

A wearable device has been developed in this work. Five pressure sensors are attached to the bottom of the insole sheet to receive the force exerted on the foot while walking. The wearable device can perform real-time prediction of the gait cycle regarding seven phases—stand, heel strike, foot flat, midstance, heel off, toe-off, and swing—using the kNN algorithm. The experiment consisted of slow walking (1–2 km/h), resulting in 90–100% prediction accuracy; normal walking (3–5 km/h), resulting in 85.14–97.43% prediction accuracy; and fast walking (6–8 km/h), resulting in 30.71–70.81% prediction accuracy.

The insole device has a wireless data transceiver operating with a low delay of 15 ± 2 ms. This device uses little power and is small, lightweight, and wearable. Moreover, the proposed gait analysis technique is unaffected by the physical conditions of the wearer. Therefore, the device can be applied with a transtibial prosthesis to efficiently control it. However, there is still a limitation: walking speed. Our algorithm can support a maximum human walking speed of 6 km/h.

For future work, we will use a 3D vision system to assess the accuracy of the gait detection technique proposed in the present study and also design equipment that is more comfortable to wear.

## Figures and Tables

**Table 1:** Gait classification accuracy with k = 3.

Gait Cycle	Precision	Recall	F1-Score	Accuracy (%)
Stand	0.750	0.700	0.724	70.00
Heel Strike	0.875	0.933	0.903	93.34
Foot Flat	0.880	0.733	0.800	73.33
Midstance	0.606	0.667	0.635	66.67
Heel Off	0.846	0.733	0.786	73.33
Toe Off	0.778	0.933	0.848	93.34
Swing	1	1	1	100.00

## Data Availability

Not applicable.
